# Joint Meeting of the Indiana and Illinois Veterinary Medical Associations

**Published:** 1890-07

**Authors:** H. R. Macaulay

**Affiliations:** Secretary


*Joint Meeting of the Indiana and Illinois Veterinary Medical Association.*—The joint meeting of the Indiana and Illinois Veterinary Medical Association was held in Terre Haute, on the evenings of June 4th and 5th, with Dr. M. E. Knowles, president of the Indiana Association, in the chair. The mayor gave an address of welcome.

After the minutes had been read and adopted and preliminary business disposed of, Dr. W. L. Williams, president of the Illinois Association, read a paper on "The Pathology of Azoturia as Suggested by its History and Symptoms."

The essayist first dwelt upon the essential surroundings necessary to cause the trouble. The active life preceding a period of rest for at least twenty-four hours when confined by halter, and that if confined for a period extending over ten days the liability to contract the disease was greatly lessened, the condition of the animal having a great deal to do with predisposition.

The writer then takes up the different theories of the trouble as explained especially by Williams, Robertson, Bollinger, Frohner and Dieckerhoff.

Professor Williams accounts for the disease by stating that the blood before exercise contains great quantities of albumen, and that exercise by oxydation transforms this albumen into urea, hippuric acid, etc., with which the blood is overloaded and the kidneys stimulated to excrete what is deleterious.

Professor Robertson, in "Equine Medicine," advocates the doctrine of hyperalbuminosis, and believed that the pathological products of albuminoid decomposition began to accumulate in the blood prior to exercise, but does not attempt to go further.

Bollinger terms the disease "Toxæmic hæmoglobinuria," resulting from action of cold or exercise, forming toxic ferment, which destroys the red blood corpuscles, causing hæmoglobinuria.

Frohner terms the disease "rheumatic hæmoglobinuria," due to taking cold, resulting in inflammation of the posterior muscles.

Dieckerhoff believes it is an acute constitutional malady with toxic ferment.

Continuing, the essayist states that as the blood is "the essential theatre of the pathological changes," it is here we must look for a solution of the trouble. Certain classes of foods produce a hyperalbuminiotic condition of the blood, which condition reaches its maximum physiological amount of this and other solids, and then gradually declines with prolonged rest, and with this declining movement the susceptibility of the animal to this trouble lessens and finally ceases. This explains part of the phenomena, but why is exercise needed to produce the disease in these cases? What changes are produced by exercise?

Professor Williams gives his theory but it is incomplete, and Dieckerhoff's theory does not appear right, or why does the toxic ferment cease its action with rest?

In continuing the examination of the blood, the essayist states that after a comparatively brief rest the water in the blood lessens to increase again with prolonged rest, so that in a suitable case for azoturia the animal's blood has a minimum amount of water and a maximum amount of albuminoids, and that "any augmentation of either of these strained physiological states produce at once a pathological condition."

The essayist accounts for the muscles over the loins and hips becoming the first and most pronouncedly affected, by their being the great locomotor agents, and as they do the greatest amount of work the blood supply to them is greatly increased, and as the changes in the blood constituents occur, blood stasis takes place, causing muscular cramp followed by paralysis.

The writer remarks that by following up this last view of the disease we have a safe guide to prevention by properly exercising, feeding, etc. It also suggests the most successful means of treatment—rest and quietude. The essayist does not believe in drastic purges, as they further draw upon the water in the blood and tissues; nor does he advocate sinapisms, blisters or irritating applications of any sort to any part of the body, and in a nervous animal would not pass catheter in earlier stages of disease.

In the discussion following the paper Dr. Ferling inquired of essayist if he thought there was any predisposition? No.

Dr. Bell inquired if the animal was more subject to a return of the trouble after having been once attacked? The essayist would say no, although there have been many cases that have had the trouble two or three times.

Dr. Thompson inquired if it was more frequently seen in draft horses. The essayist considered not, although he could not speak from experience, his practice being almost entirely among draft stock. Dr. Mylne: Is change of blood local or general? Local in gluteal regions, because of their doing most work. Dr. Knowles: Has season any influence? Yes, because of care, and the work is more irregular in Spring.

Dr. Macaulay: The cases he has observed were almost entirely in the Spring, and, because of this, was inclined to think the terminal nerve endings and the sudoriferous glands played an important part. On a fit subject for the trouble coming from the stable to the cooler air the sensitive nerve endings become affected and the sweat glands close and do not throw off their share of what is deleterious in the system, and thus one great channel of excretion is closed, and, because of this, other channels, chiefly kidneys and liver, are called upon for greater exertion; but being unable to take from the blood what is detrimental as fast as it is manufactured by tissue waste, the blood becomes surcharged with urea and other allied products, and the disease issues. In almost every case the information is that after going a short distance, the animal commenced trembling and broke out into a profuse perspiration, the sweat glands acting only after the disease is noticed to be present. Dr. Ferling: How long does an animal generally remain down, and yet recover? Nine or ten days. Those remaining down longer generally succumb. Dr. Diggs mentioned a case that had been down for nine weeks, and recovered. Dr. Diggs: What percentage of cases recover after going down, in your practice? From 60 to 75 per cent. Do you not think a nervous horse more liable to the trouble? Yes, because of faster driving at the time of starting. Dr. Curphey: Would not the use of a very soft catheter prevent excessive nervousness? The essayist thought it would, but would use no catheter whatever for three or four hours.

Dr. Franklin: What treatment do you advise? Quietness, no catheter, enemas, and, if necessary, oil—sometimes stimulant. Dr. Buckner: How does essayist quiet animal? A good strong man at head is as good as anything; often uses gelsemium or chloral. Cases naturally have cessation of pain in three or four hours without any medicine.

Dr. Mylne next read a paper on the "Surgical Treatment of Fistula." The essayist dwelt on the various locations of fistulæ, their structure and usual causes, before passing on to treatment. In all cases get to bottom of sinus by surgery. Use antiseptics and keep animal where it can be under personal observation. The essayist gave very minute details both as to operating and the use of anæsthetics, advising, as far as possible, for incisions to be in direction of natural lay of hair, to prevent, if possible, any cicatrix, and while free in surgery, yet be as conservative as possible; always have dependant openings. Treatment by seton, actual cautery and corrosive injections was also handled.

*Discussion.*—Dr. Bell mentioned a case of fistula into cæcum following puncture—all coming out of fistula five days after puncture. Used injection of corrosive sublimate, and had case well in four days. Dr. Williams mentioned a case of brood mare with abscess on cervical vertebra, which had never been treated, although ugly scars were present. This ended fatally.

Dr. Paul Paquin, State Veterinarian of Missouri, then gave an extemporaneous lecture on bacteria, and mentioned results of experiments made by him in preventive inoculation for Texas fever, etc. The doctor mentioned that in order to understand the life of bacteria—these microbes which exist in the borderland between the animal and vegetable kingdoms—one must look into and study the life of higher animals and plants. Look at the life of the sugar beet, for instance, and we find that it stores away sugar in its roots, which remains there dormant, as it were, until time of blossoming, when it becomes soluble, is absorbed and causes the flowering. The active agent in this metamorphosis is termed diastase. Animals live on plants, and in the assimilation of the one into the body of the other we find many changes taking place, such as conversion of starch into sugar, etc. The agent in this change is diastase. Take any kind of microbe, the commonest, perhaps, being the one of decomposition. We find changes in the dead body, such as liquefaction, the agent for this change also being diastase. This would show that in many things the lines are parallel. All have to change things before assimilation, and without these bacteria, which cause these changes, there would be no life.

What is the difference between microbes which cause disease and those that do not? In action none; they all act by changing substances on which they live. The real difference is, some live on live animals and others on ferments. Some live better in fluids, as blood; some on tissues. The septicæmia microbe transforms the blood for its nourishment, and thus causes the disease. In tuberculosis the microbe lives better on tissues, and they transform the tissues where they live. All diseases are due to living causes, and microbes are not the product of disease, as was once supposed. The lecturer then spoke shortly on tuberculosis—which causes one-eighth of the deaths in this country—of glanders and charbon, and then went minutely into the study of Texas fever. The micro-organism of this disease is not very readily isolated. Dr. Billings was the first man who found germs in the livers of animals affected with the trouble and connected them with the disease. Others had seen them, but he first grasped their importance. It is now known that these germs can be transmitted, by inoculation, from Southern calves unborn to Northern cattle. It is not anthracoid; the changes in the blood are quite different. We know that Texas cattle remain quite healthy, while Northern cattle contract the disease, and that these Northern cattle cannot transmit the disease to other Northern cattle. Why are these things so? The cattle in the South are "naturally" inoculated before birth, as the lecturer has proved, having found the germ in diseased calves before their birth. This is nature's method of prevention. Now, when inoculation means prevention, why cannot Northern men inoculate their cattle before shipping South?

The lecturer then went on to describe his experiments in this line, and how his last results from use of cultivated germs were very satisfactory. Forty head of short horns and thirty Herefords were shipped South for stock purposes, along with others not inoculated. Of those inoculated none were lost; of the others about 75 per cent. In Texas fever the blood is not fluidified as in anthrax, but by a using up of red blood corpuscles. The disease is, to a large extent, of blood corpuscles themselves. The softness of spleen is due to a severe congestion, but there are more microbes in liver than in spleen. How is it Northern cattle do not transmit to other Northern? Because the germ of Texas fever requires a resting period, during which it regains its vigor, and until such time as vigor is regained it lies dormant.

On motion of Dr. Curphey, seconded by Dr. Bell, the meeting was adjourned until next evening.

The morning of the 5th was spent in an enjoyable and profitable manner by visiting some of the largest stock farms in the vicinity of the city. In the evening, at 8 P.M., the meeting was called to order, and Dr. G. Ferling read his paper on "Antefebrin, or Acetanilid." The essayist first mentioned how the drug was manufactured, and then detailed a number of cases that he had successfully treated, both for influenza, strangles and rheumatism, and one case of lanunitis. In exhibiting the drug, the essayist always combined it with some stimulant, such as whiskey or digitalis, to counteract the depressing influence the drug has on the heart when given alone. The essayist gave doses of ʒiv, and repeated every four hours, and in almost all cases had a considerable diminution of temperature after the second dose. Externally, the writer had used the drug on unhealthy sores, which generally improved under this treatment, the acetanilid forming a coating over them.

In the discussion following, Dr. Bell mentioned that he had used the drug in much larger doses—ʒvi doses three times daily. Dr. Williams had used it advantageously in pink eye, and gave ℥i dose every twelve hours; this generally reduces the temperature before twenty-four hours. Could only recall one case of heart complication, and had used it in conjunction with nitrous ether, giving the latter in ℥i doses every three hours. Had found acetanilid to control action of heart rather than interfere with it.

Dr. Knowles—Does the drug cut the course of pink eye short?

The essayist thought not. Dr. Williams thought it did, but was not prepared to say that it actually did.

Dr. Bell--How does antifebrin reduce temperature?

The essayist said it was by its action on the heart; while Dr. Bell thought it was more likely due to some power it possessed of destroying the fever-producing microbe.

Dr. Knowles inquired of essayist if he had ever tried good nursing and no medication in cases where temperature was high. No. Dr. Knowles then mentioned a case of single pneumonia where temperature was 108°. He gave three doses of the drug, of ℥j each, without any change. He stopped giving medicine, and horse improved.

Dr. Buckner then read his paper on "Pneumonia." After mentioning the various causes, the essayist spoke of the changes incident in the lungs, and divided the disease into three stages, the first stage appearing with the rigors and ending with consolidation. A post-mortem examination of this condition discovers lung in a condition of inflammatory œdema. The second stage is that of pulmonary or red hepatization. The lung in this stage is heavy and solid to the feel. On section, it presents a red granular surface, which readily breaks down. The third stage is one of resolution, when the temperature lowers. The exuded product undergoes molecular degeneration, the fibrinous elements becoming completely emulsified and the corpuscles more or less broken down by fatty degeneration. Pneumonia terminated in resolution, abscesses, gangrene and a chronic pneumonia.

In discussing the symptoms the essayist mentioned that in early stages it was difficult to say what the trouble was to be—whether pneumonia or pleurisy—but by the third or fourth day there should be no mistake. Patient standing: pulse from 70 to 60; temperature from 104°-106°, with accelerated breathing, etc.

In treatment the essayist believed in being guided by the symptoms as they arose. Had found vesication over chest very beneficial, having a tendency to abort lesions and protect against pleurisy. Put in good hygienic surroundings and give stimulants and tonics or sedatives, according to case.

Dr. Knowles inquired if essayist thought there was much use in medicating? No; more could be done by giving patient best of hygienic surroundings. Dr. Bell: Does quinine, antipyrine, etc., help the disease. Yes; by stimulating.

Dr. Gustmeyer, M.D., one of Terre Haute's ablest physicians, mentioned some cases of pneumonia in the horse that he had seen some years ago; how that while kept in the stable where they had contracted the disease numbers died, but on being removed to a field where good air was obtainable all those that were then afflicted recovered.

Dr. Thompson moved a vote of thanks to Drs. Paquin and Williams. Carried unanimously.

Dr. Williams being connected with the *American Veterinary Review*, made an appeal for the complete papers read before the Indiana Association, in return for which the *Review* will give the Association reprints of their meetings at a nominal price.

Moved by Dr. Macaulay, seconded by Dr. Buckner, that Dr. Williams' proposition be accepted. Carried.

Following this there was the naming of essayists for next meeting, and because of the United States Veterinary Association having its meeting in Chicago next September, it was moved by Dr. Thompson, seconded by Dr. Ferling, that the next meeting of the Indiana Association be held in Indianapolis, on the first Wednesday in January next. Moved by Dr. Buckner, seconded by Dr. Bell, that the next meeting be commenced on Wednesday night and continued nntil Thursday morning and evening. Carried. Moved by Dr. Mylne, seconded by Dr. Diggs, that a vote of thanks be accorded Drs. Knowles and Thompson for their courtesy on Thursday morning. Carried.

On motion of Dr. Macaulay the meeting then adjourned.

H. R. MACAULAY, *Secretary.*

